# Unsupervised content-preserving transformation for optical microscopy

**DOI:** 10.1038/s41377-021-00484-y

**Published:** 2021-03-01

**Authors:** Xinyang Li, Guoxun Zhang, Hui Qiao, Feng Bao, Yue Deng, Jiamin Wu, Yangfan He, Jingping Yun, Xing Lin, Hao Xie, Haoqian Wang, Qionghai Dai

**Affiliations:** 1 Department of Automation, Tsinghua University, Beijing, 100084 China; 2 Tsinghua Shenzhen International Graduate School, Tsinghua University, Shenzhen, 518055 China; 3 Institute for Brain and Cognitive Sciences, Tsinghua University, Beijing, 100084 China; 4 School of Astronautics, Beihang University, Beijing, 100191 China; 5 Beijing Advanced Innovation Center for Big Data and Brain Computing, Beihang University, Beijing, 100191 China; 6 Department of Pathology, Sun Yat-sen University Cancer Center, Guangzhou, 510060 China; 7 State Key Laboratory of Oncology in South China, Guangzhou, 510060 China; 8 Collaborative Innovation Center for Cancer Medicine, Guangzhou, 510060 China; 9 Beijing Innovation Center for Future Chips, Tsinghua University, Beijing, 100084 China

**Keywords:** Microscopy, Biophotonics, Imaging and sensing

## Abstract

The development of deep learning and open access to a substantial collection of imaging data together provide a potential solution for computational image transformation, which is gradually changing the landscape of optical imaging and biomedical research. However, current implementations of deep learning usually operate in a supervised manner, and their reliance on laborious and error-prone data annotation procedures remains a barrier to more general applicability. Here, we propose an unsupervised image transformation to facilitate the utilization of deep learning for optical microscopy, even in some cases in which supervised models cannot be applied. Through the introduction of a saliency constraint, the unsupervised model, named Unsupervised content-preserving Transformation for Optical Microscopy (UTOM), can learn the mapping between two image domains without requiring paired training data while avoiding distortions of the image content. UTOM shows promising performance in a wide range of biomedical image transformation tasks, including in silico histological staining, fluorescence image restoration, and virtual fluorescence labeling. Quantitative evaluations reveal that UTOM achieves stable and high-fidelity image transformations across different imaging conditions and modalities. We anticipate that our framework will encourage a paradigm shift in training neural networks and enable more applications of artificial intelligence in biomedical imaging.

## Introduction

Deep learning^1^ has enabled great progress in computational imaging and image interpretation^2,3^. As a data-driven methodology, deep neural networks with high model capacity can theoretically approximate arbitrary mappings from an input domain to an output domain^4,5^. This capability offers a promising solution for image transformation, which is one of the most essential applications in biomedical imaging. The purpose of image transformation is to convert one type of image into another to either highlight important information or gain access to previously inaccessible information. The information extraction and prediction processes performed during such transformation are beneficial for biomedical analyses because they make otherwise imperceptible structures and latent patterns visible.

Recently, several network architectures have been employed for image transformation. U-Net^6^, one of the most popular convolutional neural networks (CNNs), has been demonstrated to show good performance in cell segmentation and detection^7^, image restoration^8^, and 3D fluorescence prediction^9^. Some elaborately designed CNNs can also perform image transformation tasks such as resolution improvement^10^ and virtual fluorescence labeling^11^. Moreover, Generative Adversarial Network (GAN), an emerging deep learning framework based on minimax adversarial optimization in which a generative model and an adversarial discriminative model are trained simultaneously^12,13^, can learn a perceptual-level loss function and produce more realistic results. GANs have been verified to be effective for various transformation tasks, such as super-resolution reconstruction^14,15^, bright-field holography^16^, and virtual histological staining^17^.

The superior performance of current deep learning algorithms strongly depends on substantial high-quality training data. In conventional supervised learning, vast amounts of images and corresponding annotations are necessary. However, manual annotation tends to be time-consuming and error-prone, especially for image transformation tasks requiring pixel-level registration. Although data augmentation and transfer learning have been widely employed to reduce the necessary training data scale, collecting even a small number of aligned image pairs still requires hardware modifications and complicated experimental procedures. In some cases, strictly registered training pairs are impossible to obtain because of the fast dynamics of the biological activities of interest or the incompatibility of the relevant imaging modalities. In essence, the conflict between the indispensability of annotated datasets and the dearth of paired training data obstructs the advancement of deep learning in biomedical imaging. At present, the invention of Cycle-consistent GAN (CycleGAN) has made the unsupervised training of CNNs possible^18^. CycleGAN can transform images from one domain into another without requiring paired data and exhibits performance comparable to that of supervised methods. This framework has been used in style transfer for natural images^19–21^ and in medical image analysis^22–24^. For optical microscopy, a few forward-looking studies have utilized CycleGANs to remove coherent noise in optical diffraction tomography^25^ and for the segmentation of bright-field images and X-ray computed tomography images^26^.

To enhance the feasibility of unsupervised learning in biomedical applications, we here propose an Unsupervised content-preserving Transformation for Optical Microscopy (UTOM). Through the introduction of a saliency constraint, UTOM can locate image content and ensure that the saliency map remains almost unchanged when performing cross-domain transformations. Thus, distortions of the image content can be avoided, and semantic information can be well preserved for further biomedical analyses. Using this method, we implemented in silico histological staining of label-free human colorectal tissues with only unpaired adjacent sections as the training data. We also demonstrated the application of UTOM for fluorescence image restoration (denoising, axial resolution restoration, and super-resolution reconstruction) and virtual fluorescence labeling to illustrate the capability and stability of the proposed transformation.

## Results

### Principle of UTOM

**Fig. 1:**
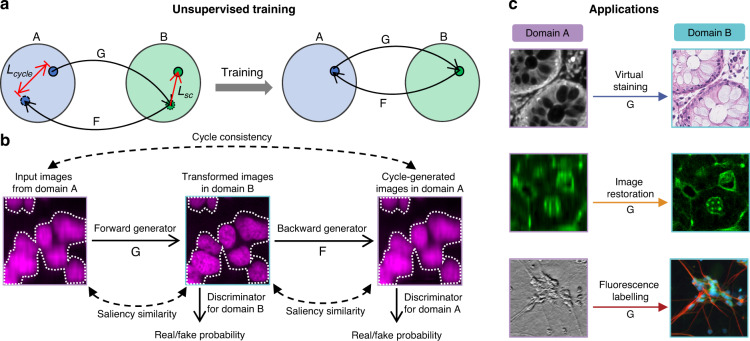
Principle of UTOM. **a** Two image sets, A and B, represent two image domains. No paired training data are required in these two sets. A forward GAN, G, and a backward GAN, F, are trained simultaneously, each to learn one direction of a pair of opposite mappings. A cycle-consistency constraint (*L*_*cycle*_) and a saliency constraint (*L*_*sc*_) are enforced to guarantee invertibility and fidelity, respectively. After proper training, reversible mappings between the two domains can be learned and memorized in network parameters. **b** The unfolded flowchart of UTOM. Input images from domain A are first transformed into the modality of domain B by the forward generator and then mapped back to domain A by the backward generator. Two discriminators are simultaneously trained to evaluate the quality of the mappings by estimating the probability that the transformed images were obtained from the training data rather than the generators. The cycle-consistency constraint is imposed to make the cycle-generated images as close to the input images as possible. The saliency constraint enforces the similarity of the image saliency (annotated by white dotted lines) before and after each mapping. **c** Once UTOM converges, the forward generator can be loaded to perform domain-transformation tasks such as virtual staining, image restoration, and fluorescence labeling

A fundamental schematic of UTOM is depicted in Fig. 1a. Two image sets (A and B) are first collected to sample the source domain and the target domain. These two image domains belong to different modalities (*e.g*., bright field and fluorescent, low SNR and high SNR, anisotropic resolution and isotropic resolution, etc.). No pre-aligned image pairs are required in these two image collections. Thus, images in the source domain and the target domain can be acquired independently. A forward GAN and a backward GAN are trained simultaneously to learn a pair of opposite mappings between the two image domains. Along with the cycle-consistency loss^18^, a saliency constraint is imposed to correct the mapping direction and avoid distortions of the image content. For each domain, a discriminator is trained to judge whether an image was generated by the generator or was drawn from the training data. When the network converges, the two GANs reach equilibrium^27^, which means that the discriminators cannot distinguish images produced by their generators from the training data. An image can then be mapped back to itself through sequential processing by both generators, and more importantly, the saliency map maintains a high similarity after each transformation (Fig. 1b). For subsequent applications, the pre-trained forward generators will be loaded, and images never before seen by the network will be fed into the model to obtain corresponding transformed images (Fig. 1c).

### In silico histological staining through unsupervised training

First, we validated UTOM on transformation from label-free autofluorescence images to standard hematoxylin and eosin (H&E)-stained images. Clinically, H&E-stained histopathology slides provide rich information and are widely used for tumor diagnosis^28^. However, conventional histopathological imaging involves many complicated steps, thus hindering intraoperative diagnosis and fast cancer screening. Consequently, the ability to generate standard H&E-stained images from images acquired via label-free imaging methods has long been pursued by biomedical researchers^29–31^. Autofluorescence images of unstained tissue can reveal histological features, and the transformation from autofluorescence images to H&E-stained images is helpful for supporting pathologists in the diagnosis process. Conventional supervised methods for virtual histological staining^17^ rely on laborious sample preparation and imaging procedures, which are not suitable for collecting large datasets for training clinical-grade computer-assisted diagnosis systems. UTOM can overcome these limitations by breaking the dependence on pixel-level registered autofluorescence-H&E training pairs.

**Fig. 2:**
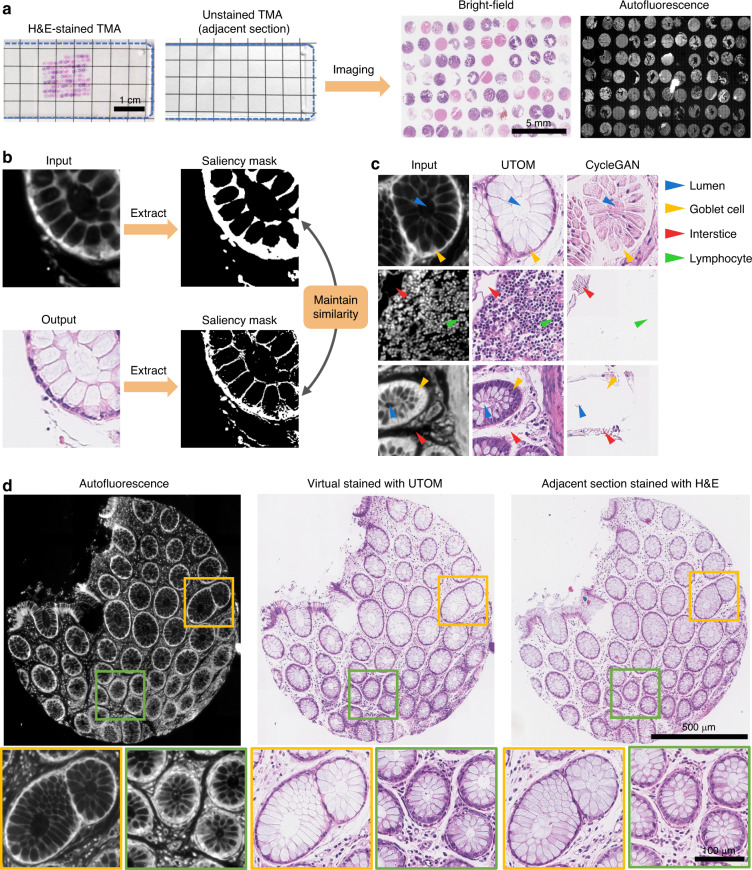
UTOM enables in silico histological staining of label-free colorectal slides without paired training data. **a** Sections of colorectal tissues were assembled into TMAs. H&E staining was performed for tissue sections adjacent to sections for which the staining procedure was skipped. The scale bar represents 1 cm. Bright-field imaging and fluorescence imaging were performed for H&E-stained and label-free TMAs, respectively. The scale bar represents 5 mm. **b** Benefiting from the saliency constraint, UTOM maintains the similarity of saliency masks when images are transformed from the autofluorescence domain to the standard H&E staining domain. **c** UTOM exhibits superior convergence and stability. Histological structures can be preserved after transformation, and distortion of the image content can be effectively avoided. The arrowheads indicate various histological features, including lumens (blue arrowheads), goblet cells (yellow arrowheads), interstices (red arrowheads), and lymphocytes (green arrowheads). **d** Left: Autofluorescence image of a label-free tissue core. Middle: H&E staining predicted by UTOM. Right: Bright-field image of the corresponding adjacent H&E-stained section for comparison. With UTOM, the glandular architectures are well preserved, and the cellular distributions are clearly histologically resolved. The scale bar for the overall images represents 500 μm; the scale bar for the enlarged images represents 100 μm

We extracted samples from human colorectal tissues. After formalin fixation, paraffin embedding, and sectioning, adjacent sections were separated for label-free processing and H&E staining processing. We assembled the tissue sections into tissue microarrays (TMAs) with tens of independent cores (see the Methods). Then, we performed bright-field imaging of the H&E-stained TMAs and autofluorescence imaging of the label-free TMAs (Fig. 2a). In the whole-slide bright-field and autofluorescence images, tissue cores in the same position were adjacent sections with similar but not identical histological features. The H&E-stained adjacent sections were used as the reference to evaluate the quality of the transformation.

To construct the training dataset (representing the autofluorescence domain and the H&E-stained domain), we randomly split the whole-slide images from each domain into approximately 7000 tiles of 512 × 512 pixels. Such a small input size can reduce the memory requirements and accelerate the training process. Then, we trained the UTOM framework to learn the mapping from autofluorescence to standard H&E staining. The saliency constraint in UTOM ensured that the extracted saliency map of the input autofluorescence image remains similar when transformed to the H&E domain (Fig. 2b), which endows this framework with superior stability and reliability. Without this constraint, the semantic information of the autofluorescence image would be lost, and the output H&E-stained image would be distorted (Fig. 2c and Fig. S1). This is detrimental to clinical diagnosis and could lead to severe consequences. After content-preserving transformation with UTOM, typical histological features such as lumens, interstices, goblet cells, and lymphocytes are well preserved and become legible to pathologists. To test the transformation of large images, we partitioned them into multiple tiles with 25% overlap and then stitched the predicted tiles together to obtain the final results (see the Methods). A tissue core virtually stained by UTOM is shown in Fig. 2d. Glandular architectures and nuclear distributions can be stained in silico with high fidelity, achieving an effect comparable to that of real H&E staining. In addition to visual inspection, we trained a CNN for gland segmentation (see the Methods) to quantitatively evaluate whether UTOM staining would affect the accuracy of downstream segmentation tasks. Segmentation based on UTOM-stained images achieved almost the same accuracy as segmentation based on real H&E-stained images (Fig. S2), indicating that UTOM can accurately reconstruct H&E-stained structures from autofluorescence images and effectively preserve the rich histopathological information provided by such images.

### Fluorescence image restoration

**Fig. 3:**
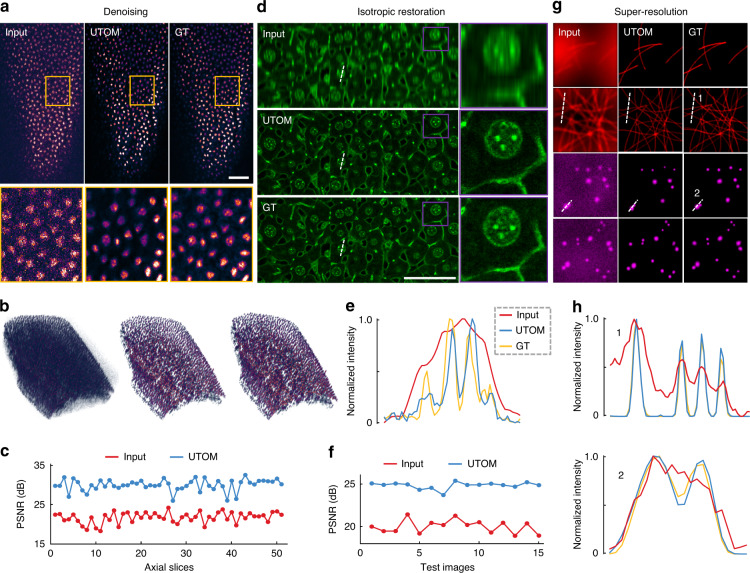
Unsupervised restoration of fluorescence images. **a** UTOM can learn to map low-SNR images to high-SNR images with high fidelity. Structures can be resolved from noisy raw data imaged at a low excitation dose. The scale bar represents 50 μm. **b** 3D visualization of a volume shows that some unrecognizable details become clearly visible after denoising. From left to right are the raw images, images restored with UTOM and the corresponding ground truth (GT). **c** PSNRs of 51 axial slices before and after denoising, improved by ~8 dB on average. **d** Degradation of axial resolution can be restored by UTOM without relying on paired training data. The scale bar represents 50 μm. **e** Intensity profiles along dashed lines in **d**. Aliasing peaks in the input image can be separated by UTOM. **f** PSNRs of 15 test images before and after isotropic restoration, improved by ~5 dB on average. **g** UTOM can be used to resolve sub-diffraction structures such as microtubules and secretory granules from widefield images. **h** Intensity profiles along the dashed lines in **g**. Structures that cannot be recognized according to the Rayleigh criterion become resolvable again

Next, we applied UTOM for the restoration of fluorescence images based on the experimental data released by Weigert et al^8^. In this series of applications, both the input and the output images were single-channel greyscale images. The target of UTOM was to learn to restore the degraded information in the input images. For the denoising of confocal images of planaria^8^, we first generated the training set by randomly partitioning large images from each domain (low-SNR and high-SNR images) into ~18,000 small 128 × 128 tiles (see the Methods). No paired images were included in the two training sets. Then, we trained UTOM on this dataset to learn the transformation from the low-SNR domain to the high-SNR domain. Some low-SNR images that had never previously been seen by the network were then used to evaluate our model, and the results are shown in Fig. 3a. Our results show that UTOM can transform low-SNR images into high-SNR images while maintaining the original cell structures. We also visualized a 3D volume composed of 2856 (7 × 8 × 51) tiles to demonstrate the generalization ability of our model (Fig. 3b). The enhancement after denoising is remarkable, and some details that are unrecognizable in the original noisy volume become clearly visible. For quantitative analysis, we calculated the peak signal-to-noise ratios (PSNRs) of the images before and after transformation and found that the image PSNR was increased by ~8 dB (Fig. 3c). Furthermore, we compared the performance of UTOM with that of 3D CARE^8^, a state-of-the-art supervised method for fluorescence image restoration (Fig. S3a), and found that the UTOM results have more authentic intensity distributions (Fig. S3b).

The axial resolution degradation of optical microscopy can also be restored by our unsupervised learning framework. We trained UTOM on two unpaired image sets generated from a mouse liver dataset^8^ following the same procedure as for denoising. As shown in Fig. 3d, an accurate mapping for axial resolution restoration was established after proper training. The blurring caused by the axially elongated point spread function (PSF) was restored, and some aliasing structures in the original images were successfully resolved (Fig. 3e). We quantified the network performance by calculating the image PSNR, as shown in Fig. 3f. After UTOM restoration, the image PSNR was improved by ~5 dB on average across all 15 test images. We also verified our model on the restoration of multicolor zebrafish retina images with a different degradation coefficient to demonstrate the model capabilities (Fig. S4). Moreover, UTOM can be used to resolve sub-diffraction structures such as microtubules and secretory granules (Fig. 3g). The transformation from wide-field microscopy to super-resolution microscopy can be learned without the supervision of any paired training data. The UTOM results maintain high accuracy and fidelity and are comparable to those of the CARE network^8^ (Fig. S5). The intensity profiles of some fine structures in Fig. 3g are shown in Fig. 3h. Structures that are indistinguishable in the raw images according to the Rayleigh criterion can be recognized again after UTOM reconstruction.

### Virtual fluorescence labeling

**Fig. 4:**
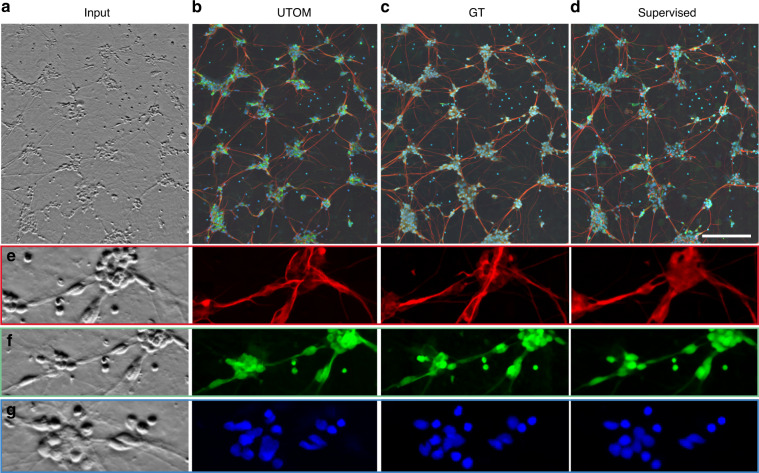
Virtual fluorescence labeling without paired training data. **a** Phase-contrast image of differentiated human motor neurons. The original image stack was average-projected for display. **b** Fluorescence images predicted by UTOM. **c** Ground-truth image acquired with a spinning disc confocal microscope. Axons, dendrites, and nuclei labeled with different fluorescence indicators are imaged in the red, green and blue channels, respectively. **d** Fluorescence images predicted by a multi-scale CNN trained on pixel-registered image pairs^11^. The scale bar represents 200 μm. **e**–**g** Enlarged views of detailed structures extracted from the red, green, and blue channels, respectively. Axons, dendrites, and nuclei can all be resolved by UTOM

Each microscopy technique has its own inherent advantages, and these advantages cannot all be achieved simultaneously because of incompatible imaging principles. Our unsupervised learning framework is particularly suitable for transformations between different imaging modalities. We applied UTOM to transform phase-contrast images to fluorescence images^11^ to enable label-free imaging with component specificity. We generated an unpaired training dataset following the same procedure as above. Each domain contained ~25,000 image tiles. The input stack, network prediction, and corresponding ground truth are shown in Fig. 4a–c, respectively. Most structures can be identified and labeled with correct fluorescence labels except for a few neuron projections. We compared our method with a conventional supervised CNN^11^ (Fig. 4d), which achieves a more realistic intensity distribution and better global effects. In this application, supervised learning shows better performance (SSIM = 0.88, averaged over 3 channels) because pixel-level aligned training pairs can make the model easier to train and guide the loss function to converge to a better solution. However, UTOM is still competitive due to its lack of reliance on paired training data, which means that images of each modality can be captured at different times, by different systems, and even from different samples. To evaluate each channel separately, three enlarged regions of interest (ROIs) are shown in Fig. 4e–g, and the structural similarity index (SSIM) was calculated to quantify the prediction accuracy for each channel (Fig. S6). Among the three channels, the blue channel has the best labeling accuracy, with SSIM = 0.87, followed by the green channel, with SSIM = 0.82. The red channel has relatively large deviations with SSIM = 0.64.

Finally, we validated the feasibility and versatility of UTOM on various tasks by benchmarking against several state-of-the-art methods, as shown in Table 1. By means of the saliency constraint, content distortions can be avoided when images are transformed between two domains. UTOM can learn correct transformations for all three applications and achieve almost the best performance among all CycleGAN-based methods. Although the identity loss shows similar performance for isotropic reconstruction and fluorescent labeling, it cannot effectively solve the problem of content distortion. The identity loss was originally designed to preserve the color composition between the input and output^18^. When the input and output have the same color composition, this can enhance the output style to some extent. In other words, UTOM can solve the problem of content distortion but requires parameters for saliency segmentation. The identity loss is a parameter-free constraint, but its effectiveness is not guaranteed for all tasks.

**Table 1:** Benchmarking of UTOM against state-of-the-art methods

	Histological staining	Isotropic reconstruction		Fluorescence labeling	
Metrics	Expert inspection	SSIM	PSNR	SSIM	PSNR
Supervised CNN	×	0.75	26.3	0.89	24.7
CycleGAN	N	0.25 ± 75.7%	19.5 ± 9.6%	0.76 ± 2.8%	19.1 ± **1.9%**
CycleGAN with identity loss	N	0.70 ± 3.2%	24.9 ± 3.0%	0.77 ± 1.6%	19.6 ± 2.9%
UDCT	N	0.23 ± 78.8%	19.3 ± 9.6%	0.77 ± 2.2%	**20.1** ± 3.3%
UTOM	**Y**	**0.71** ± **1.6%**	**25.1** ± **1.5%**	**0.78** ± **0.5%**	20.0 ± 2.1%

We compared UTOM with other transformation methods, including state-of-the-art supervised CNNs^8,11^ and several variants of CycleGANs^18,26^. Three tasks (*i.e*., *in silico* histological staining, isotropic reconstruction, and virtual fluorescence labeling) were taken as examples. Due to the lack of paired ground-truth images, three pathology experts were invited to judge the histological staining performance. Here, ‘ × ’ means that the task could not be completed with the corresponding method, ‘N’ indicates incorrect transformation with distorted image contents, and ‘Y’ indicates correct transformation with preserved image contents. The qualitative judgments of the three experts were consistent with each other. Considering the convergence problem of CycleGAN, each CycleGAN-based method was trained five times. The mean values and relative errors of each metric are shown here (bold text indicates the most significant values)

## Discussion

In summary, we have demonstrated an unsupervised framework (UTOM) to implement image transformations for optical microscopy. Our framework enables content-preserving transformations from a source domain to a target domain without the supervision of any paired training data. By imposing a saliency constraint, UTOM can locate the image content and ensure that the saliency map remains similar when transformed to another domain. This improvement can effectively avoid distortions of the image content and disordering of semantic information, leading to greatly enhanced stability and reliability and clearing obstacles hindering biomedical analysis. We verified this unsupervised learning framework on several tasks of image transformation between different imaging conditions and modalities, such as in silico histological staining, fluorescence image restoration, and virtual fluorescence labeling. We evaluated the quality of transformed images by performing downstream tasks or comparing them to corresponding ground-truth images. Quantitative metrics show that our unsupervised learning framework can learn accurate domain mappings and achieves comparable performance to some state-of-the-art supervised methods. Significantly, the lack of reliance on any registered image pairs during the training process distinguishes UTOM from other competing methods. Laborious image acquisition, annotation, and pixel-level registration procedures are no longer necessary.

The proposed method has the potential to accelerate a shift in the training paradigm of deep neural networks from conventional supervised learning to an unsupervised approach. In optical microscopy, unsupervised learning has unique advantages, especially when a sample is undergoing fast dynamics or preparing paired data would cause destruction of the sample. Moreover, incorporating the physics of image formation into either the forward or backward mapping of CycleGAN is helpful for reducing the number of network parameters and, more importantly, improving the quality of transformation^24,32^, which is a promising strategy when the physics of image formation is explicit. It can be expected that once the reliance on paired training data is eliminated, more applications of deep learning in optical microscopy will be made possible.

## Materials and methods

### TMA preparation and image acquisition

Tissues were extracted surgically or endoscopically and were prepared as formalin-fixed and paraffin-embedded (FFPE) samples. Using a tissue array instrument (Minicore Excilone, Minicore), FFPE blocks were punched and tissue cores (approximately 1.2 mm in diameter) were removed with a hollow needle. These tissue cores were then inserted into recipient holes in a paraffin block and arranged into a uniform array. The re-embedded tissues were subsequently sliced into 3-μm sections, and adjacent sections were separated and mounted on different glass slides. We performed H&E staining on one slide and left the adjacent slide unstained. For convenience of visual inspection, tissue cores placed in the same position on the label-free slide and the corresponding H&E-stained slide were adjacent sections.

For the autofluorescence imaging of label-free TMAs, we used the fluorescence imaging mode of a commercial slide scanner microscope (Axio Scan.Z1, Zeiss) equipped with a 40x/0.95 NA objective (Plan Apochromat, Nikon). We chose the DAPI excitation light source and the corresponding fluorescence filter packaged with the scanner. The imaging and acquisition process (including ROI selection, field-of-view switching, autofocusing, image stitching, etc.) was controlled automatically by the bundled software (Zen, Zeiss). Bright-field images of H&E-stained TMAs were captured using the bright-field mode of the slide scanner.

### Loss function

To ensure a reliable transformation from domain A to domain B, the mapping between the source domain and the target domain should be reversible to establish a one-to-one correspondence. A cycle-consistency loss^18^ was used to guide the two GANs to form a closed cycle by quantifying the difference between the original images and the cycle-generated images:

1 $$\begin{array}{l}L_{cycle}(G,F) = {\mathbf{E}}_{a\sim p_{data}(a)}\left[ {\left\| {F\left( {G(a) - a} \right.} \right\|_1} \right]\\ + {\mathbf{E}}_{b\sim p_{data}(b)}\left[ {\left\| {G(F(b)) - b} \right\|_1} \right]\end{array}$$

Here, **E** represents element-wise averaging, and *a* and *b* are instances from domain A and domain B, respectively. *G* and *F* are the forward generator and the backward generator, respectively. The identity loss in the original CycleGAN model, which was designed to preserve the tint of input images, was abandoned because it was unhelpful for preserving the image content (Fig. S1).

Additionally, we imposed a saliency constraint on the loss function to realize content-preserving transformation. This constraint is based on the observation that, unlike the backgrounds of natural scenes, the backgrounds of optical microscopy images have similar intensities. For instance, the background of fluorescence images is black, while that of bright-field images is white. Using a threshold segmentation method, the regions corresponding to salient objects can be roughly extracted. Accordingly, the saliency constraint was designed to maintain the consistency of the content masks extracted via threshold segmentation:

2 $$\begin{array}{l}L_{sc}(G,F) = {\mathbf{E}}_{a\sim p_{data}(a)}\left[ {\left\| {T_\alpha (a) - T_\beta (G(a))} \right\|_1} \right]\\ + {\mathbf{E}}_{b\sim p_{data}(b)}\left[ {\left\| {T_\beta (b) - T_\alpha (F(b))} \right\|_1} \right]\end{array}$$

where $T_\alpha$ and $T_\beta$ are segmentation operators parameterized by thresholds *α* and *β*, respectively. In our implementation of the saliency constraint, we manually selected the thresholds for saliency segmentation. This process included three steps: (1) We first selected a background region and computed the background intensity as the average of all pixels in that background region. (2) Then, we selected a foreground region and computed the foreground intensity as the average of all pixels in that foreground region. (3) The segmentation threshold was finally computed as the average value of the background intensity and the foreground intensity. Threshold segmentation is a rough estimation of salient objects, and the selection of segmentation thresholds is relatively flexible. Selecting thresholds using the built-in ‘Threshold’ function of ImageJ is also feasible. We recommend checking the thresholds manually before training to ensure that the desired image content can be segmented. The Heaviside step function for thresholding was approximated by a sigmoid function to preserve non-trivial derivatives, i.e.,

3 $$\begin{array}{l}T_\alpha (x) = {\mathrm{sigmoid}}\left[ {100(x - \alpha )} \right]\\T_\beta (x) = {\mathrm{sigmoid}}\left[ {100(x - \beta )} \right]\end{array}$$

It is worth noting that regardless of the task, the image content should be mapped to a value of 1, while the background should be mapped to a value of 0. For virtual histopathological staining, pixel intensity=0 indicates the background in domain A, while pixel intensity=255 indicates the background in domain B. The segmentation operator for domain B was adjusted to

4 $$T_\beta {\mathrm{(}}x{\mathrm{)}} = {\mathrm{1}} - {\mathrm{sigmoid}}\left[ {100(x - \beta )} \right]$$

More details and some real-data examples are shown in Fig. S7 and Fig. S8. Finally, the full loss function can be formulated as follows:

5 $$\begin{array}{l}L_{cycleGAN} = L_{GAN}(G) + L_{GAN}(F)\\ + \lambda [L_{cycle}(G,F) + \rho L_{sc}(G,F)]\end{array}$$

where $L_{GAN}(G)$ and $L_{GAN}(F)$ are the adversarial losses of the forward GAN and the backward GAN, respectively (Supplementary Notes), and *λ* and *ρ* are hand-tuned weights to adjust the relative strength of the cycle-consistency loss and the saliency constraint. The influence of *ρ* is investigated in Fig. S9. Inspired by the adaptive learning rate in modern gradient descent optimizers, *ρ* is designed to exponentially decay throughout the training progress. The saliency constraint is imposed only at the beginning of the training process to guide the network to converge in a better direction, and the roughly estimated saliency maps guide the network to generate objects at correct locations. During the later stage of training, *ρ* will converge to zero, and the saliency maps will not interfere with the fine adjustment of the image details.

### Network architectures and training details

The generator and discriminator of UTOM adopt the classical architecture of the CycleGAN model (Supplementary Notes and Fig. S10). Considering the diversity of microscopy images, many image transformation tasks involve changing the number of channels of the image, and thus, the two GANs must have matching input and output channel numbers. For the generator, the kernel size of the first convolutional layer was set to 7 to endow this layer with large receptive fields for the extraction of more neighborhood information. We used padded convolutional layers to keep the image size unchanged when passing through convolutional layers. All the downsampling and upsampling layers were implemented by means of strided convolution with trainable parameters. For the discriminator, we adopted the PatchGAN^33,34^ classifier. This architecture penalizes structures at the scale of local patches to encourage high-frequency details. More specifically, it divides the input image into small patches, and classification is performed at the patch scale. The ultimate output is equivalent to the average of the classification loss on all patches.

The Adam optimizer^35^ was used to optimize network parameters. The exponential decay rates for the 1st and 2nd moment estimates were set to 0.5 and 0.999, respectively. We used a batch size of 1 and trained our network for ~10k iterations. We used graphics processing units (GPUs) to accelerate computation. On a single NVIDIA GTX 1080 Ti GPU (11 GB memory), the whole training process for a typical task (single-channel images, 128 × 128 pixels, 50k iterations) took approximately 10 h. Considering the possibility of using multiple GPUs for parallel computation, the training time can be further reduced.

### Data pre- and post-processing

Autofluorescence images were first converted into 8-bit TIFF files using a MATLAB (MathWorks) script for consistency with the H&E-stained images (8-bit RGB files). Although the dynamic range was narrowed, the contrast of 8-bit input files was sufficient for virtual histological staining. In addition, using 8-bit autofluorescence images can lower storage requirements and accelerate the processes of data reading, writing, and transmission. After file conversion, tissue cores were cropped out separately from whole-slide images and divided into a training set and a test set. Then, the large images were randomly split into 512 × 512 tiles for training. For data pre-processing of fluorescence image restoration and virtual fluorescence labeling, we used 16-bit files for both the input and output images. Each dataset with ground truth was divided into a training part and a testing part at a ratio of approximately 5:1. For each image pair in the training part, we randomly decided whether to select the original image or its ground truth with a probability of 0.5 and discarded the other image. Then, the selected images were collected into two subsets corresponding to domain A and domain B. This operation guaranteed that there were no aligned image pairs in these two image sets (Fig. S11).

In the testing phase, all original images in the test set were fed into the pre-trained forward generator. Large images were partitioned into small tiles with 25% overlap. For post-processing of transformed images, we cropped away the boundaries (half of the overlap) of the output tiles. Stitching was performed by appending the remaining tiles immediately next to each other (Fig. S12). 3D visualization was performed with the built-in *3D viewer* plugin of *ImageJ*. Reconstructed data volumes were adjusted to the same perspective for convenience of observation and comparison.

### Gland segmentation

To reduce the amount of data required for training, the segmentation of colorectal glands was performed based on transfer learning. We first pre-trained a standard U-Net model on the GlaS dataset^36,37^ to learn to extract generic features of colorectal glands. However, we found significant differences between the tint of our H&E-stained slides and those in the GlaS dataset. To improve the segmentation accuracy, we manually annotated a few images and then fine-tuned the model on our dataset. Data augmentation was adopted in both the pre-training phase and the fine-tuning phase to reduce data requirements. After training, large images were partitioned into 512 × 512 tiles with 25% overlap and then fed into the pre-trained model. The predicted tiles were stitched together to form the final results. For quantitative analysis, the intersection-over-union (IoU) scores between network-segmented masks and corresponding manually annotated masks were computed^38^.

### Benchmarks and evaluations

We compared our unsupervised framework with state-of-the-art supervised CNNs and several other unsupervised methods to demonstrate its competitive performance. The supervised models were optimally selected for benchmarking. We used the original CARE network^8^ for the task of fluorescence image restoration. For planaria denoising, we adopted the packaged TensorFlow implementation of CSBDeep, and the model was configured and trained in 3D. For isotropic restoration and super-resolution reconstruction, we also retrained the CARE network using a training set of a similar size to that of UTOM. All images were organized in 2D in these two tasks. For each experiment that required retraining the network, a hold-out validation set was prepared to monitor the training process to avoid overfitting. In the virtual fluorescence labeling experiment, an elaborately designed multi-scale CNN trained on pixel-level aligned image pairs^11^ was loaded, and test images were fed into and flowed through the network. The released models are sufficiently reliable to produce the best results that a supervised network can achieve. For in silico histological staining, three pathology experts were invited to judge the correctness of the transformations due to the lack of paired ground-truth images.

The evaluation strategy was based on not only investigating the differences visually but also providing quantitative analyses of transformation deviations. The quantitative metrics we used for image-level evaluation were PSNR and SSIM. SSIM is suitable for measuring high-level structural errors, while PSNR is more sensitive to absolute errors at the pixel level. For RGB images, SSIMs were averaged over the three channels. For the task of denoising, we also calculated the histograms of the pixel values using *ImageJ* to show the distributions of image intensity (Fig. S3). To better visualize the transformation deviations, we assigned the network output and the corresponding ground truth to different color channels, i.e., the network output to the magenta channel and the ground truth to the green channel. Because magenta and green are complementary colors, if the structures before and after transformation were in the same position and had the same pixel values, these structures in the merged image would appear white. Otherwise, each corresponding pixel would be displayed as the color with the larger pixel value (Fig. S6). This strategy was used to independently visualize the transformation errors (especially position offsets) across different channels in virtual fluorescence labeling.

## Supplementary information

Supplementary Information

## Data Availability

The dataset for histological staining is available from the corresponding author upon request. The data for fluorescence image restoration are available at https://publications.mpi-cbg.de/publications-sites/7207/. The dataset used for virtual fluorescence labeling can be found at https://github.com/google/in-silico-labeling/blob/master/data.md. Some data processed with our pre-processing pipeline that can be used for training and testing have been made publicly available at https://github.com/cabooster/UTOM/tree/master/datasets.

## Code availability

Our PyTorch implementation of UTOM is available at https://github.com/cabooster/UTOM. The scripts for data pre- and post-processing have also been uploaded to this repository.
