# Supplementary material for: Unsupervised content-preserving transformation for optical microscopy
Source: Light Sci Appl. 2021 Mar 1;10:44. doi: 10.1038/s41377-021-00484-y (PMC7921581; doi:10.1038/s41377-021-00484-y)
Supplement: Supplementary file 1 — Supplementary Information [file 41377_2021_484_MOESM1_ESM.pdf]

Supplementary Information

Unsupervised content-preserving transformation for optical microscopy

Xinyang Li[1,2,3†], Guoxun Zhang[1,3†], Hui Qiao[1,3†], Feng Bao[1,3], Yue Deng[4,5], Jiamin Wu[1,3], Yangfan He[6,7,8], Jingping Yun[6,7,8], Xing Lin[1,3,9], Hao Xie[1,3], Haoqian Wang[2,3*] & Qionghai Dai[1,3*]

[†]*These authors contributed equally to this work.*

*Correspondence: qhdai@mail.tsinghua.edu.cn, wanghaoqian@tsinghua.edu.cn

Supplementary Figures

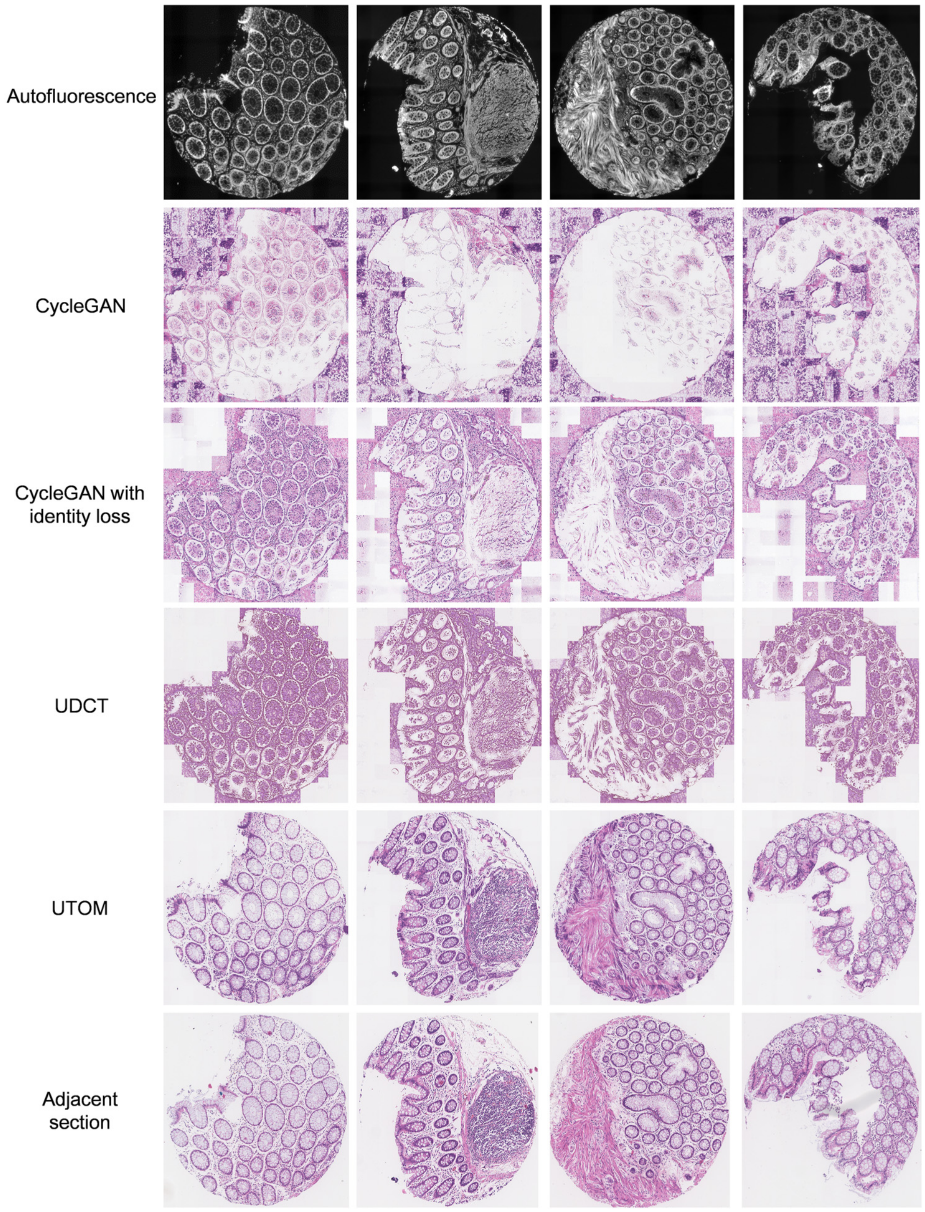

Figure S1 | UTOM can preserve the image content during transformation. Current unsupervised methods do not have the content-preserving ability and the image content is distorted when transformed to the target domain. With the saliency constraint, UTOM can learn content-preserving transformations and the semantic information can be well maintained. Adjacent sections stained with haematoxylin and eosin (H&E) are shown in the bottom row for reference.

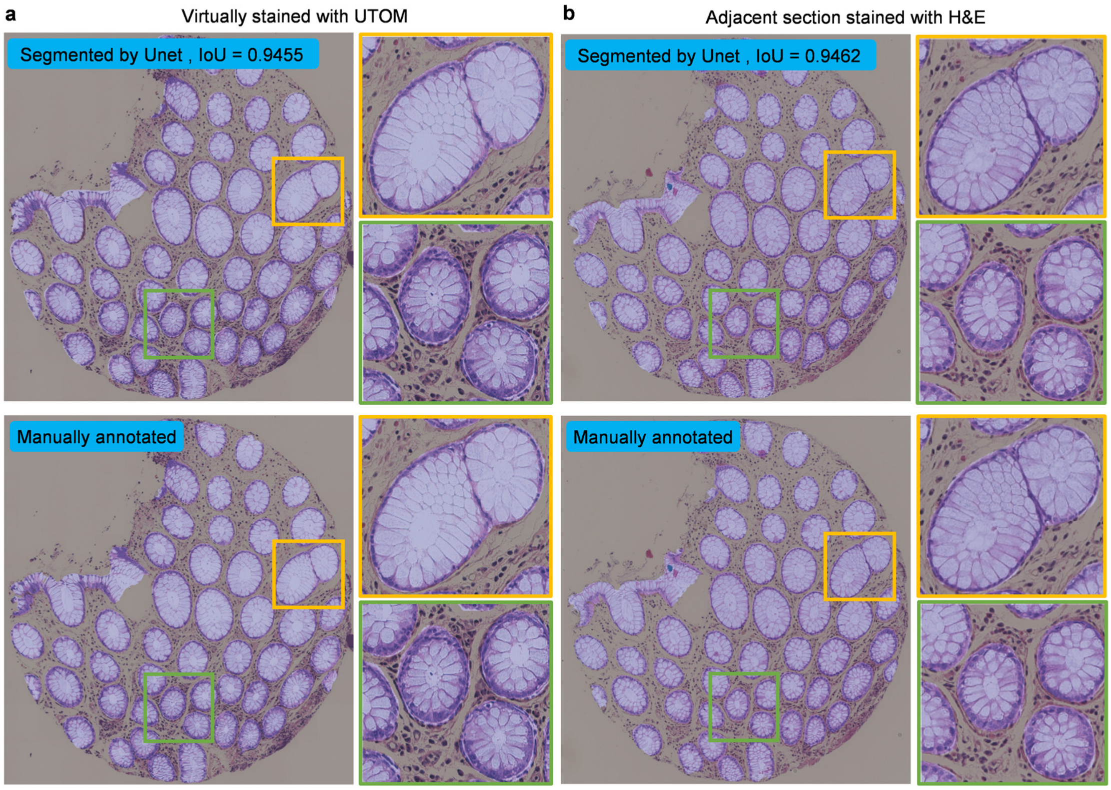

Figure S2 | Gland segmentation for UTOM-stained and H&E-stained slides. A U-Net was trained to segment secretion glands form histological images. **a**, Segmentation of UTOM-stained slide (IoU=0.9455). **b**, Segmentation of corresponding H&E-stained adjacent section (IoU=0.9462). Segmentation masks were highlighted in bright purple. Manually annotated masks (bottom panel) serve as the ground truth.

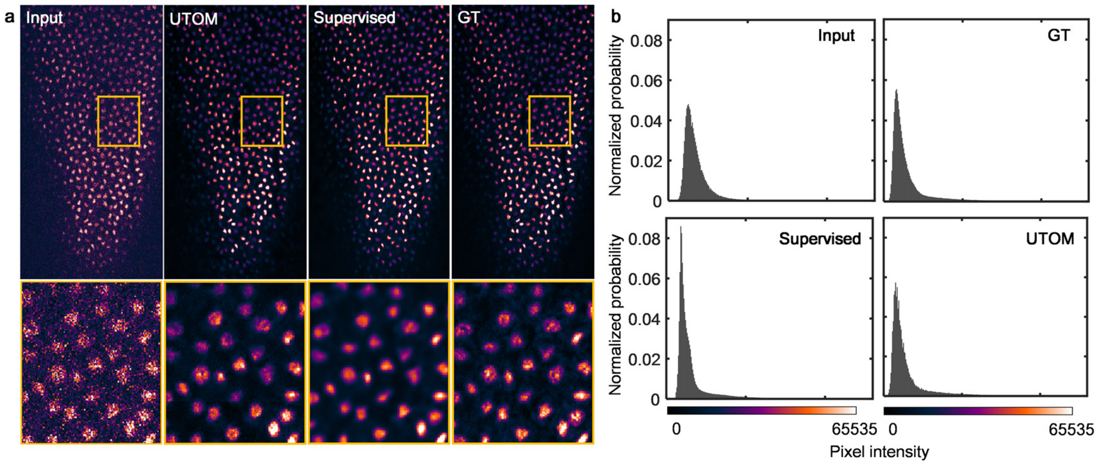

Figure S3 | High-fidelity denoising with UTOM. **a**, The result of UTOM, the ground truth, and the result of supervised CARE network are shown for comparison. **b**, Histograms revealing the distributions of pixel intensity. The histogram of UTOM result is more approximate to the ground truth (GT) than that of the supervised method, indicating a better preservation of intensity distribution.

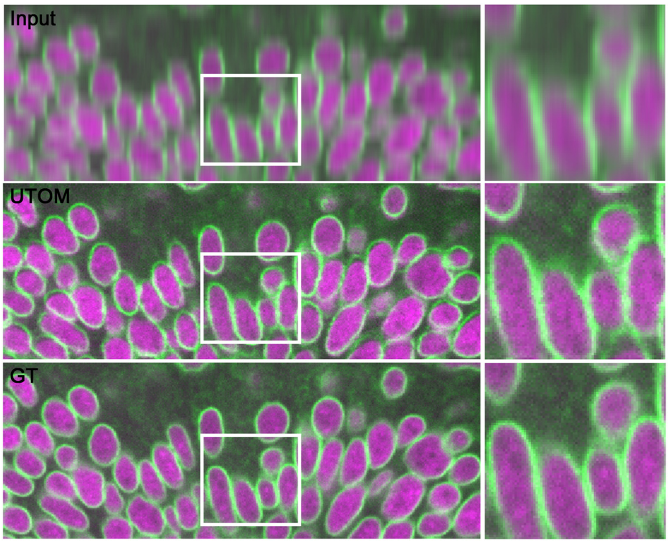

Figure S4 | Isotropic restoration of degraded axial resolution on zebrafish retina dataset. Nuclei and nuclear envelopes were labelled with DRAQ5 (magenta) and GFP-LAP2b (green), respectively. Our method can restore the degraded axial resolution.

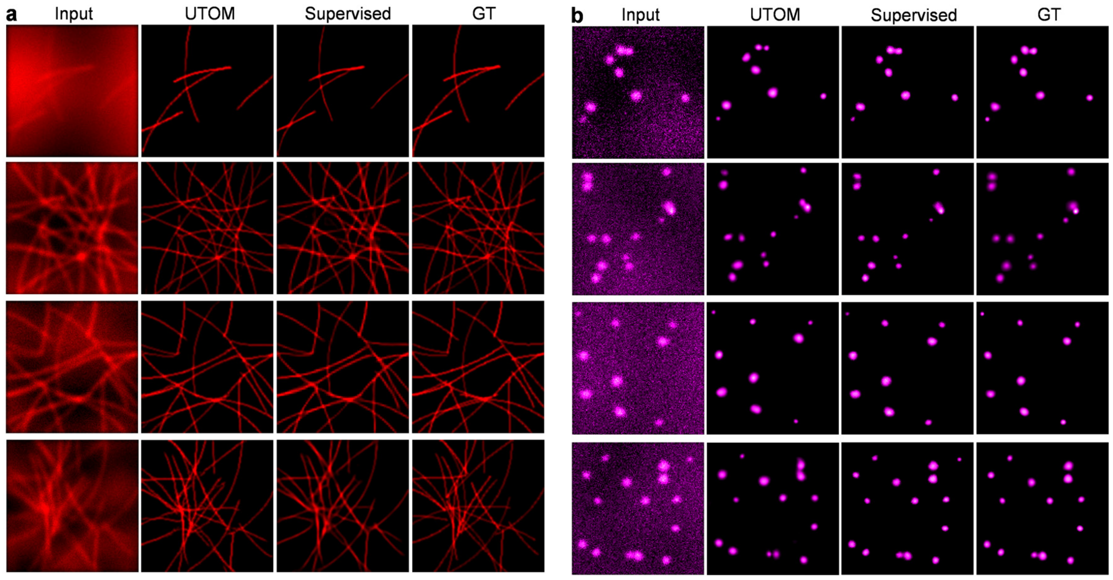

Figure S5 | High-fidelity super-resolution reconstruction by UTOM. Sub-diffraction structures such as **a**, microtubules and **b**, granules can be resolved from wide-field images. The original input images, the results of UTOM, the results of 2D CARE network, and corresponding ground-truth images are shown in each column.

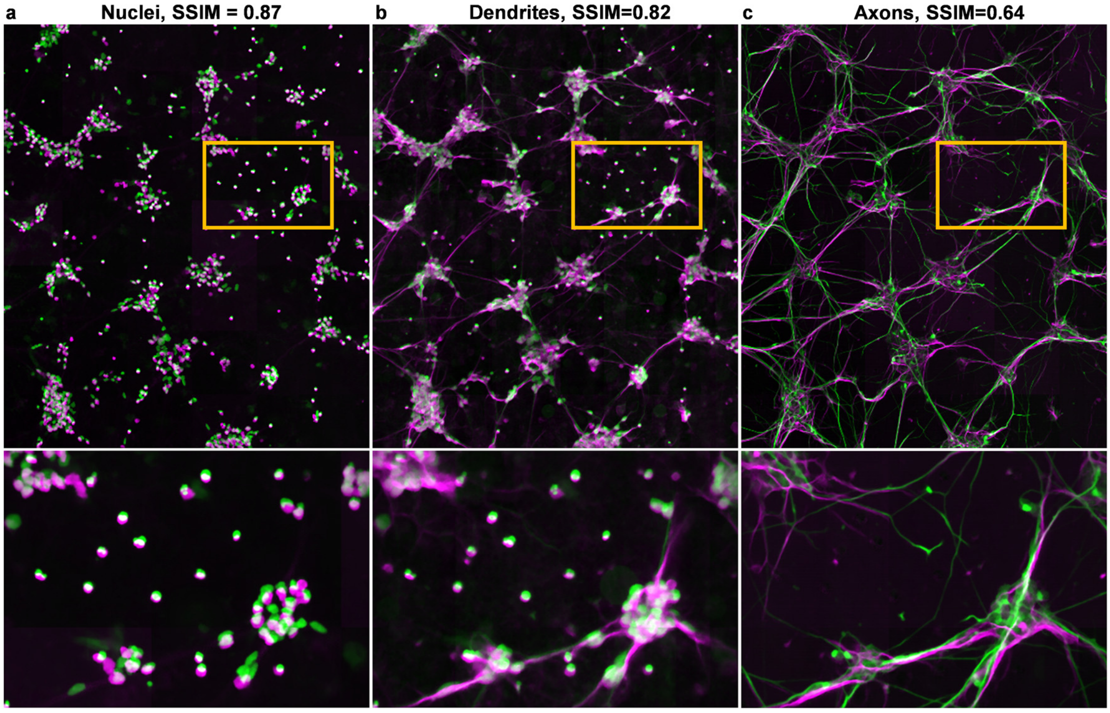

Figure S6 | Merged maps of virtual fluorescence labelling. For each channel, the results of UTOM (magenta) and corresponding ground-truth images (green) were merged together. **a**, The blue channel that labels the nuclei of human motor neurons. **b**, The green channel that labels the dendrites. **c**, The red channel that labels the axons. Images in the bottom row are enlargements of the boxed regions.

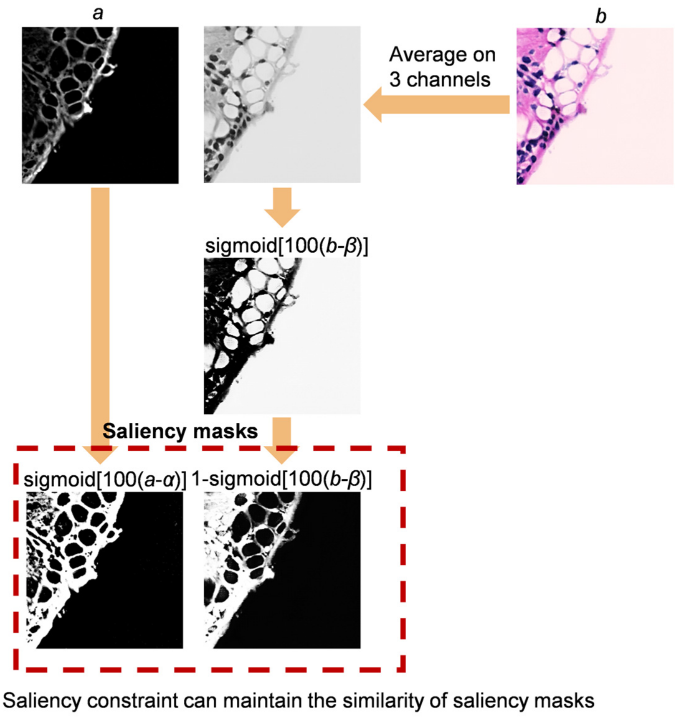

Figure S7 | Saliency constraint in virtual histopathological staining. For domain A (autofluorescence images), saliency masks were extracted directly by sigmoid[100(a-α)]. For domain B (H&E images), the RGB images were first converted to its greyscale version by averaging the three channels. Then, the saliency masks were extracted by 1-sigmoid[100(b-β)]. The image content should be mapped to 1 and the background should be mapped to 0.

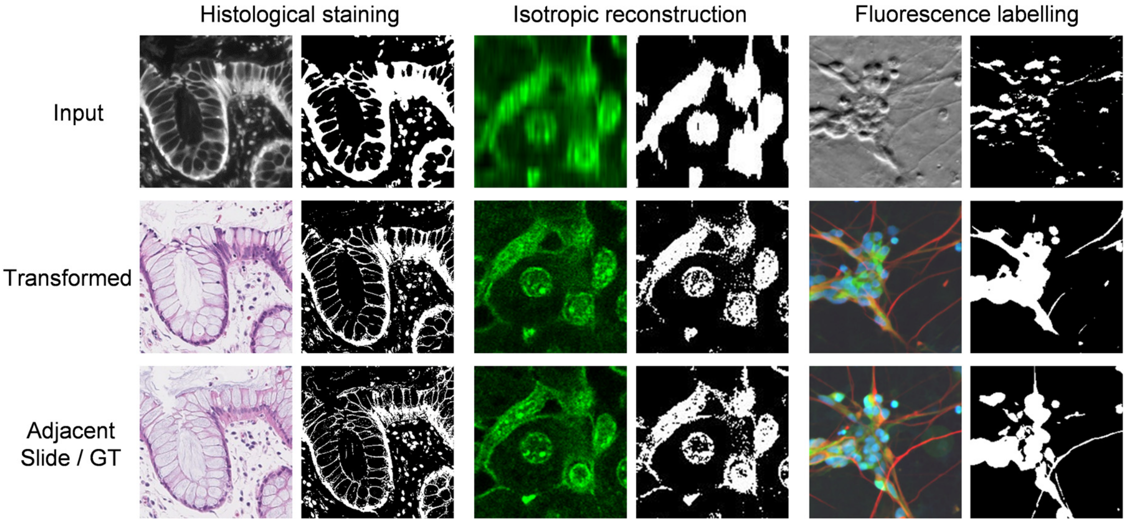

Figure S8 | Visualization of saliency maps. Saliency maps extracted by the saliency constraint in histological staining, isotropic reconstruction, and fluorescence labelling. The saliency constraint can highlight the locations of objects (*i.e.*, cells, nuclei, stroma, *etc.*) and keep them nearly unchanged when transformed from one domain to the other.

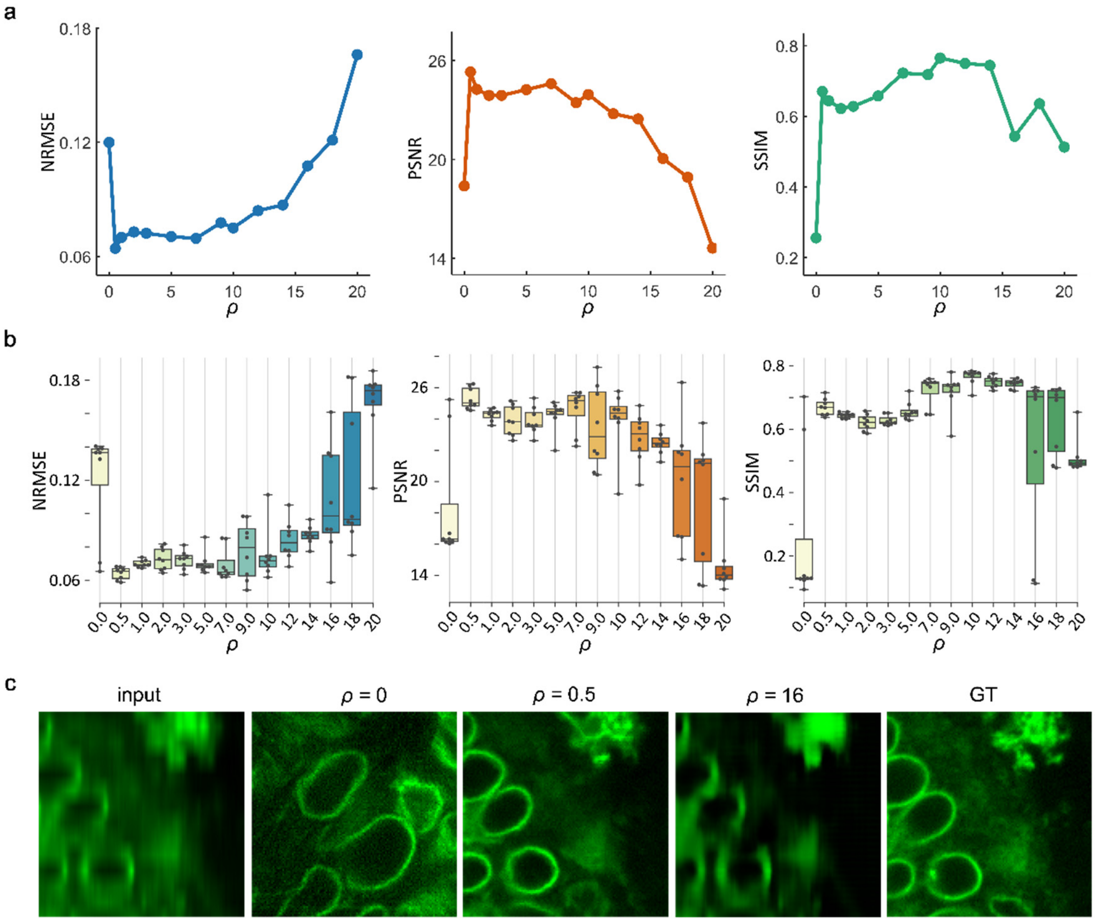

Figure S9 | Performance of UTOM when the saliency constraint was imposed with different constant weights. a, Averaged NRMSE/PSNR/SSIM of 8 independent experiments (each ρ) on the zebrafish retina dataset (Fig. S4). The saliency constraint was imposed with different constant weights ranging from 0 to 20. For each experiment, NRMSE/PSNR/SSIM were arithmetically averaged on all 144 image patches in the test set. **b**, Box-dot plots show the distributions of NRMSE/PSNR/SSIM obtained with different ρ. **c**, Typical results under different ρ. Without saliency constraint, the network was unstable and sometimes converged to wrong mappings. The saliency constraint can effectively correct the mapping bias. However, when constant ρ is relatively large, other terms will be less important and the performance will degrade.

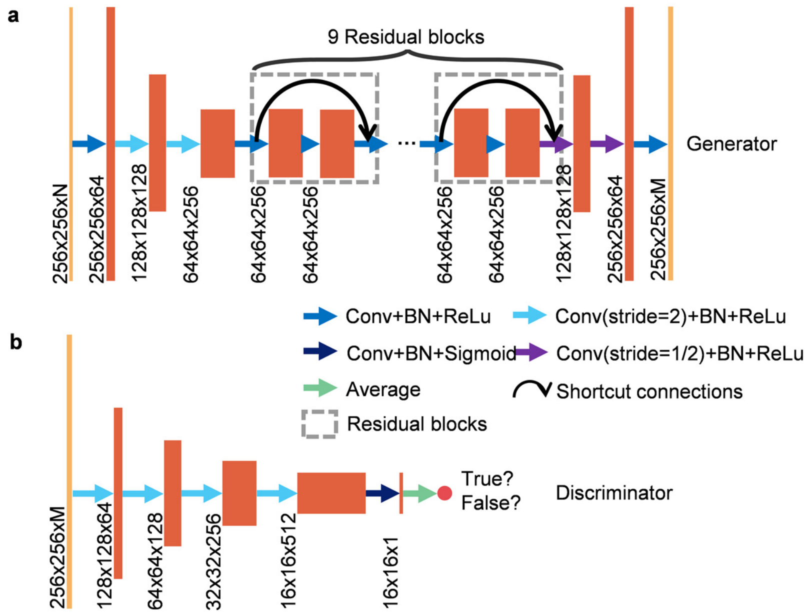

Figure S10 | Network architectures. Here we take 256×256 input size as an example. Each coral rectangle represents a feature map extracted by corresponding convolutional kernels. **a**, The generator is a multi-layer residual network with downsampling input layers and upsampling output layers. **b**, The discriminator (PatchGAN classifier) uses multiple strided convolution for abstract representation. It generates a matrix, in which each element corresponds to a patch in the input image. The ultimate output is the average of the loss over all patches.

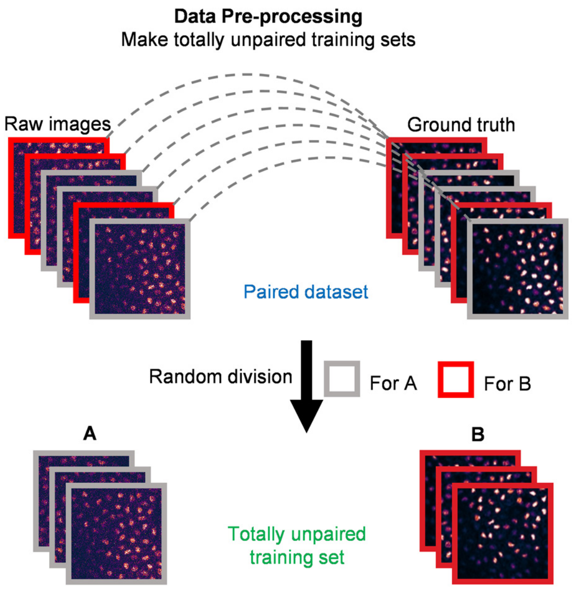

Figure S11 | Data pre-processing pipeline. Some of our training sets were from published datasets with paired ground-truth images. We randomly selected one half of the dataset and collected its raw images into domain A, and then selected the other half of the dataset and collected its ground-truth images into domain B.

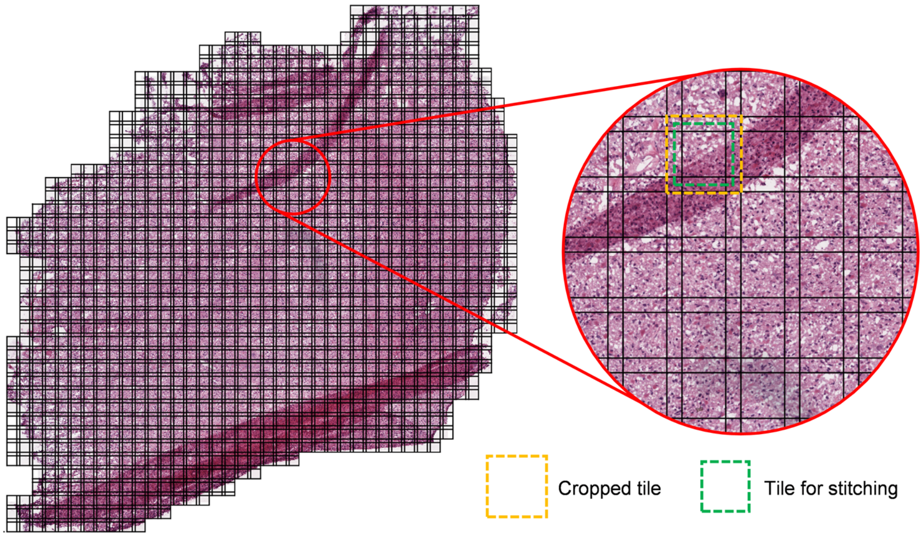

Figure S12 | Tiling and stitching in pre- and post-processing. In most cases, images needed to be transformed are extremely large in pixel size. In our data processing pipeline, large images were partitioned into multiple overlapping tiles to reduce memory requirements and improve training efficiency. The edges of output patches were cut out and the rest parts were stitched together to form a large image.

Supplementary Notes

Network architectures and the loss function.

UTOM is composed of two GANs to learn the mapping between two image domains in an unsupervised manner. The architectures of the generator and the discriminator are visualized in Fig. S10. The first three layers of the generator are downsampling layers implemented by strided convolution to extract low-level abstract representations. Nine stacked residual blocks are followed to extract high-level features. The number of residual blocks reflects the model capacity. More residual blocks are recommended for more complex tasks. The last three upsampling layers are also implemented by strided convolution. They are used to integrate extracted features and rescale the image to its original size. The discriminator is a relatively shallow CNN. Each layer downsamples the feature maps but doubles the channel number. The last convolution layer generates a single-channel feature map and classification is performed on each element of this feature map (PatchGAN classifier). The final true or false label is generated by averaging individual labels of all elements. Each convolution layer in both the generator and the discriminator contains a nonlinear activation unit. Whether to use the sigmoid function or rectified linear unit (ReLU) is marked with corresponding arrows in Fig. S10.

It is worth mentioning that the input and output channel numbers of the two generators should match to ensure that they can form a complete cycle, especially when images in domain A and those in domain B have different channel numbers. In terms of the objective function, the first part is the frequently-used adversarial loss, which can be formulated as the most common form:

$$\mathcal{L}_{GAN}(G)=\mathbf{E}_{b\sim p_{data}(b)}\left[\log D_B(b)\right]+\mathbf{E}_{a\sim p_{data}(a)}\left[\log(1-D_B(G(a))\right]$$
$$\mathcal{L}_{GAN}(F)=\mathbf{E}_{a\sim p_{data}(a)}\left[\log D_A(a)\right]+\mathbf{E}_{b\sim p_{data}(b)}\left[\log(1-D_A(G(b))\right], \quad \text{(S1)}$$

where D_A and D_B represent the discriminator of the forward GAN and the backward GAN, respectively. Lowercase letters a and b are images from the domains represented by corresponding uppercase letters. **E** is the expectation operator.

The second part of the loss function is the cycle-consistency loss, which is most essential for training the two GANs. The last part is the saliency constraint term to correct mapping errors and improve the success rate of training. The full objective function can be formulated as

$$\begin{aligned}\mathcal{L}_{cycleGAN} = {} & \mathbf{E}_{b\sim p_{data}(b)}\left[\log D_B(b)\right]+\mathbf{E}_{a\sim p_{data}(a)}\left[\log(1-D_B(G(a))\right]+ \\ & \mathbf{E}_{a\sim p_{data}(a)}\left[\log D_A(a)\right]+\mathbf{E}_{b\sim p_{data}(b)}\left[\log(1-D_A(G(b))\right]+ \\ & \lambda\left\{\mathbf{E}_{a\sim p_{data}(a)}\left[\left\|F(G(a))-a\right\|_1\right]+\mathbf{E}_{b\sim p_{data}(b)}\left[\left\|G(F(b))-b\right\|_1\right]\right\}+ \\ & \rho\left\{\mathbf{E}_{a\sim p_{data}(a)}\left[\left\|\mathcal{T}_\alpha(a)-\mathcal{T}_\beta(G(a))\right\|_1\right]+\mathbf{E}_{b\sim p_{data}(b)}\left[\left\|\mathcal{T}_\beta(b)-\mathcal{T}_\alpha(F(b))\right\|_1\right]\right\}\end{aligned}, \quad \text{(S2)}$$

where λ is a constant to enforce cycle-consistency loss and ρ can be constant or exponentially decayed to enforce the saliency constraint. If ρ is set to be a constant, the convergence will be faster. If it is exponentially decayed, the final effect will be better because the convergence direction is only constrained at the beginning of training. Cycle consistency will not be weakened at the end of training. $\mathcal{T}_\alpha$ and $\mathcal{T}_\beta$ are segmentation operators parameterized by threshold α and β. They are used to extract saliency masks of the images in domain A and domain B, respectively. Here, we used sigmoid($100x$) to approximate the Heaviside step function of threshold segmentation to keep nontrivial gradient, *i.e.*,

$$\mathcal{T}_{\alpha}(x)\text{=sigmoid}\left[100(x-\alpha)\right],\mathcal{T}_{\beta}(x)\text{=sigmoid}\left[100(x-\beta)\right]. \quad \text{(S3)}$$

It is worth noting that no matter what the task is, the image content should be mapped to 1 while the background should be mapped to 0. For virtual histopathological staining, pixel intensity=0 means the background in domain A while pixel intensity=255 means the background in domain B. The segmentation operator of domain B should be adjusted as

$$\mathcal{T}_{\beta}(x)\text{=1-sigmoid}\left[100(x-\beta)\right]. \quad \text{(S4)}$$

More details and some real-data examples are shown in Fig. S7-S9.
